# Nonspecific increase of αβTCR^+^ double-negative T cells in pediatric rheumatic diseases

**DOI:** 10.1007/s12519-024-00854-7

**Published:** 2024-11-28

**Authors:** Kuanysh Dossybayeva, Gulsamal Zhubanova, Assel Mussayeva, Zaure Mukusheva, Aiken Dildabayeva, Galiya Nauryzbayeva, Lyudmila Akhmaltdinova, Ulbolsyn Orumbayeva, Matthew Tanko, Dimitri Poddighe

**Affiliations:** 1 Department of Medicine, Nazarbayev University School of Medicine (NUSOM), 010000 Astana, Kazakhstan; 2 Program of Pediatric Rheumatology, Clinical Academic Department of Pediatrics, University Medical Center, Astana, Kazakhstan; 3 Clinical Academic Department of Laboratory Medicine, Pathology and Genetics, Republican Diagnostic Center, University Medical Center, Astana, Kazakhstan; 4 Clinical Academic Department of Pediatrics, National Research Center for Maternal and Child Health (NRCMCH), University Medical Center(UMC), 010000 Astana, Kazakhstan; 5 College of Health Sciences, VinUniversity, Gia Lam District, Hanoi, Vietnam

**Keywords:** DNT cells, Double-negative T cells, Juvenile idiopathic arthritis, Pediatric rheumatic disorders, Systemic lupus erythematosus

## Abstract

**Background:**

An increased number of double-negative T (DNT) cells expressing the αβ T cell receptor (αβ^+^DNT cells) is one of the diagnostic criteria for autoimmune lymphoproliferative syndrome (ALPS). Moreover, these cells are expanded in a widely used murine model for lupus. However, the homeostasis of αβ^+^DNT cells remains inadequately investigated in rheumatic disorders, especially in pediatric patients.

**Methods:**

In this cross-sectional, prospective, and observational study, children with rheumatic disorders and healthy controls were recruited to analyze the quantity and characteristics of circulating DNT cells using flow cytometry.

**Results:**

Overall, the two study groups did not differ in their total DNT cell pool in the bloodstream. However, the number of αβ^+^DNT cells was significantly higher in rheumatic children than that in the controls, whereas the γδ^+^DNT cells remained similar. This expansion in the circulating pool of αβ^+^DNT cells was comparable across different rheumatic diseases, all showing significant differences from the controls in this regard. Moreover, no significant correlation was found between αβ^+^DNT cell numbers and disease activity.

**Conclusions:**

These preliminary results indicate that circulating αβ^+^DNT cells are significantly expanded in children with rheumatic disorders; however, this finding appears to be a nonspecific (disease-unrelated) marker of autoimmunity. Further and larger studies are necessary to better investigate and define the role of DNT cells in pediatric rheumatic diseases.

**Graphical abstract:**

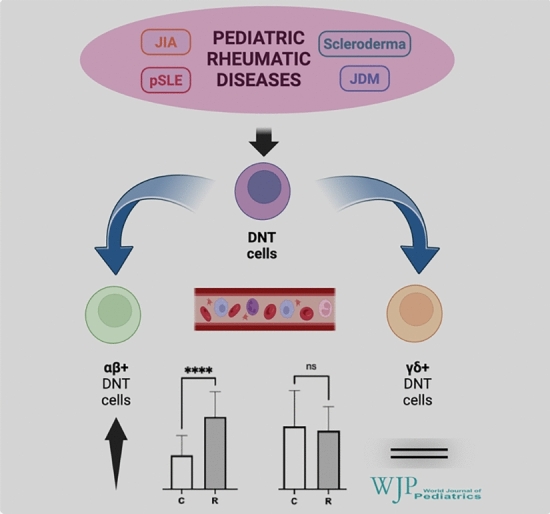

**Supplementary Information:**

The online version contains supplementary material available at 10.1007/s12519-024-00854-7.

## Introduction

Double-negative T (DNT) cells are an unconventional subset of T cells that are CD3-positive but neither express CD4 nor CD8 molecules, and lack NK cell markers [1, 2]. DNT cells can express both types of T cell receptors (TCRs), namely TCRαβ or TCRγδ; however, the former subset (αβ^+^DNT cells) has garnered more attention in clinical immunology due to its diagnostic value in autoimmune lymphoproliferative syndrome (ALPS). Indeed, circulating αβ^+^DNT cells are expanded in this disease, with counts exceeding 1.5% of total lymphocytes and/or 2.5% of CD3^+^ lymphocytes, which represent one of the diagnostic criteria for the condition [1, 3]. In addition to being characterized by reactive lymphoproliferative manifestations, such as lymphadenopathy and splenomegaly, patients with ALPS can develop several autoimmune disorders. These can include autoimmune cytopenias, systemic lupus erythematosus (SLE) or SLE-like phenotypes, and, less frequently, autoimmune nephritis, hepatitis, arthritis, and uveitis [4]. One of the most investigated murine models of spontaneous lupus, the MRL/lpr (Murphy Roths Large/lymphoproliferation gene) mouse, exhibits both lymphoproliferation and expansion of DNT cells, in addition to the development of a SLE-like phenotype that includes immune-mediated nephritis and elevated titers of autoantibodies, such as antinuclear antibodies (ANA) and anti-double-stranded DNA (anti-dsDNA) antibodies [5–7].

Unfortunately, few clinical studies have assessed DNT cells in patients affected with SLE and other rheumatic disorders, especially in children [6, 8, 9]. Therefore, the significance of DNT cells as potential biomarker and their role in the immunopathogenesis of (pediatric) rheumatic diseases have been poorly investigated and remain unclear. The present study aims to analyze the quantity and type of DNT cells in children with rheumatic diseases.

## Methods

### Study design and population

This preliminary prospective, cross-sectional, observational study aims to assess the number of circulating DNT cells in pediatric rheumatic patients compared to control children. The study period is from November 15, 2023 to March 31, 2024. Participants were recruited from the Program of Pediatric Rheumatology in the Clinical Academic Department of Pediatrics (National Research Center for Maternal and Child Health, University Medical Center, Astana, Kazakhstan), which is affiliated with the Nazarbayev University School of Medicine. Pediatric patients aged 2–17 years diagnosed with rheumatic diseases were eligible for participation, while exclusion criteria included the presence of comorbidities (beyond the primary rheumatic condition), any concomitant chronic or acute infectious diseases, and the administration of blood products within the previous six months. Control participants consisted of children without any specific diagnosis based on general pediatric evaluations.

### Data collection

Demographic data (such as age and gender), clinical information (including primary diagnosis, disease duration, disease onset, disease activity, and therapeutic regimens) and main laboratory data (including complete cell blood count and inflammatory parameters) were retrieved from patients’ medical records using a standardized data collection form created by the research team. The database containing these secondary existing data was compiled using Microsoft Excel 2022 for Mac (Version 16.68).

### Analysis of lymphocyte subpopulations

Flow cytometry analysis was used to identify and assess the main lymphocyte subpopulations, including B cells, T cells, and NK cells, as well as the lymphocyte subsets of specific interest based on the research objectives, particularly DNT cells and their αβ^+^DNT and γδ^+^DNT subsets.

**Fig. 1:**
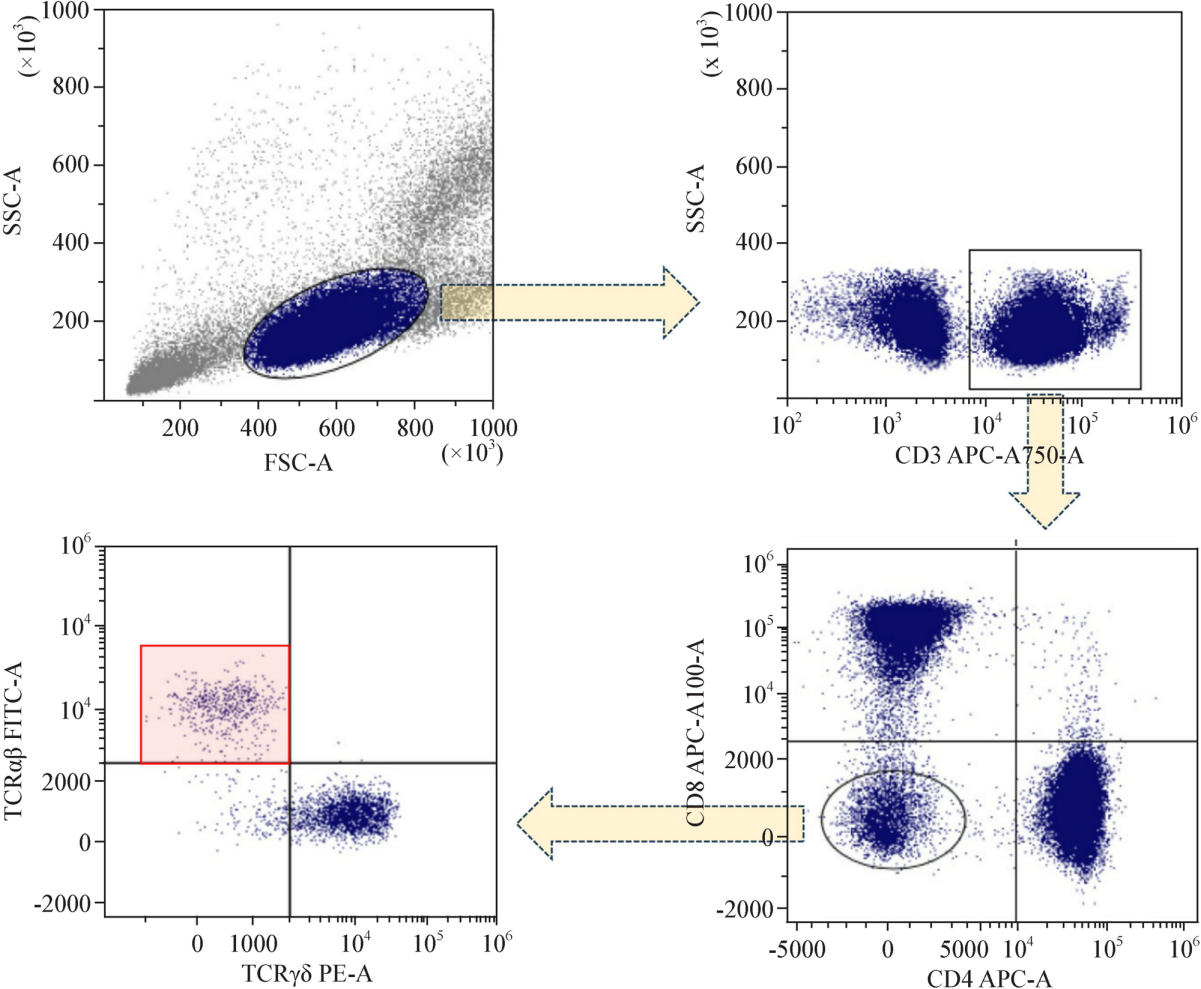
FACS gating strategy to identify and analyze DNT cells. *DNT* double-negative T, *FACS* fluorescence-activated cell sorting, *FSC* forward scatter, *SSC* side scatter, *APC* allophycocyanin, *PE* phycoerythrin, *FITC* fluorescein isothiocyanate, *TCR* T cell receptor

Peripheral blood mononuclear cells (PBMCs) were isolated from whole blood collected in EDTA (ethylenediaminetetraacetic acid) tubes. After centrifugation with Ficoll-Paque PLUS (Cytiva), PBMCs layer was incubated with Ammonium–Chloride–Potassium (ACK) lysing buffer (Gibco™, ThermoFisher). Then, the PBMCs were resuspended in staining medium buffer, composed of 10 × HBSS (Hank’s Balanced Salt Solution, Gibco™) and 1 M HEPES (N-2-hydroxyethylpiperazine-N-2-ethane sulfonic acid) (Gibco™) solution. The cell concentration was determined using an automated cell counter, in order to obtain a 1 × 10^6^ cells/mL suspension. These cells were then incubated at 4 °C for 25 min with a combination of conjugated monoclonal antibodies, including CD3-PB (UCHT1), CD4-APC (13B8.2), CD8-PC5.5 (B9.11), CD19-PE (J3-119), CD56-APC-A700 (N901), TCRγδ-PC7 (IMMU510) from Beckman Coulter (USA), and anti-TCRαβ-FITC from BD™ (USA). Subsequently, the cells were resuspended and analyzed using a DxFLEX flow cytometer (Model BE50232, Beckman Coulter, 2021). The acquired data were processed using Kaluza Analysis software (Version 2.1, Beckman Coulter, 2020). The gating strategy used to analyze DNT cell subsets is shown in Fig. 1.

### Statistical analysis

Categorical variables were expressed as absolute numbers and/or percentages, and differences between groups were analyzed using Fisher’s exact test. Continuous variables were expressed as median (Me) and interquartile range (IQR), given the non-normal distribution of values in the study groups, based on the Shapiro–Wilk normality test. Accordingly, differences between two groups were evaluated using the Mann–Whitney test, while comparisons among more than two groups were analyzed using the Kruskal–Wallis test. The potential accuracy (sensitivity and specificity) of αβ^+^DNT cell count in rheumatic patients was assessed by the area under the curve (AUC) of receiver operating characteristic (ROC) analysis. Covariation and linear regression analysis were applied to analyze the relationship between αβ^+^DNT cell count and specific variables. A *P *value < 0.05 was considered statistically significant. All statistical analyses were conducted using GraphPad Prism 9 for MacOS (Version 9.3.1).

## Results

### Demographic and clinical characteristics

A total of 110 children with rheumatic diseases were prospectively enrolled in this preliminary study. Most patients were diagnosed with juvenile idiopathic arthritis (JIA; *n* = 86; 78.2%), whereas a minor part is represented by pediatric SLE (pSLE) (*n* = 12; 10.9%) or other rheumatic disorders (*n* = 12; 10.9%). A detailed diagnostic classification of rheumatic children is described in Table 1. In addition, 40 control patients were recruited as a comparison group for the specific FACS analysis, particularly for DNT cells. The gender distribution (male/female ratio) was comparable between rheumatic and control groups (45/65 vs. 18/22; *P* > 0.05). However, the age [Me (IQR)] of controls was significantly lower than that of the rheumatic children [7.65 (3.350, 11.65) vs. 12.4 (7.25, 15.53) years; *P* < 0.001].

**Table 1:** Disease classification of rheumatic patients

Diseases	Patients, *n* (%)
Juvenile idiopathic arthritis	87 (79.1%)
Oligoarticular	31 (28.2%)
Polyarticular RF +	5 (4.5%)
Polyarticular RF-	13 (11.8%)
Psoriatic	9 (8.2%)
Enthesitis-related	16 (14.5%)
Systemic	8 (7.3%)
Undifferentiated	5 (4.5%)
Pediatric systemic lupus erythematosus	12 (10.9%)
Localized scleroderma	5 (4.5%)
Juvenile dermatomyositis	4 (3.6%)
Reactive arthritis	1 (0.9%)
Polyarteritis nodosa	1 (0.9%)

RF, rheumatic factor

Rheumatic patients were subjected to variable therapeutic regimens, including non-steroidal anti-inflammatory drugs (NSAIDs), steroid drugs (SD), conventional disease-modifying anti-rheumatic drugs (cDMARDs), and biological disease-modifying anti-rheumatic drugs (bDMARDs) (Table 2).

**Table 2:** Ongoing therapies at the time point of the blood analysis

Drugs	Patients (*n*)
Biologic DMARDs (*n* = 46, 41.8%)	
Adalimumab	18
Etanercept	15
Tocilizumab	8
Rituximab	2
Infliximab	2
Tofacitinib	1
Conventional DMARDs (*n* = 85, 77.3%)	
Methotrexate	63
Hydroxychloroquine	13
Mycophenolate mofetil	8
Sulfasalazine	1
NSAIDs (*n* = 26, 23.6%)	
Naproxen	14
Ibuprofen	6
Diclofenac	3
Aspirin	2
Lornoxicam	1
Systemic steroids (*n* = 44, 40.0%)	
Methylprednisolone	37
Prednisolone	5
Dexamethasone	2
Intra-articular steroids (*n* = 32, 29.1%)	
Triamcinolone	32

*DMARD* disease-modifying anti-rheumatic drugs, *NSAID* non-steroidal anti-inflammatory drugs

### Main hematological laboratory parameters

The complete cell blood count of children with rheumatic diseases is reported in Supplementary Table 1, where these hematological parameters are compared with those shown by controls. Notably, there is no difference in the absolute lymphocyte count between rheumatic and control patients [2.955 (2.510, 4.070) × 10^9^/L vs. 3.025 (2.293, 3.435) × 10^9^/L, respectively; *P* = 0.4515], which is important for the accurate interpretation of differences in absolute counts of specific lymphocyte subsets, including DNT cells. Among the inflammatory parameters, erythrocyte sedimentation rate (ESR) was available for the study participants. As expected, rheumatic patients showed a mild increase in ESR compared to controls [15.0 (6.5, 24.5) mm/h vs. 7.0 (4.2, 11.5) mm/h, respectively; *P* < 0.001].

With regard to the general lymphocyte subpopulations (Supplementary Table 2), rheumatic children showed an increased number of T cells with a greater expansion of CD8^+^ T cells, as also evidenced by the CD4/CD8 lymphocyte ratio [R: 1.420 (1.010, 1.780), C: 1.783 (1.410, 2.209); *P* < 0.001]. Conversely, a significant reduction of NK cells was observed in rheumatic children compared to controls. B cells were also slightly reduced in rheumatic children, which was mildly significant only when expressed as a relative (percentage) count.

### Double-negative T cells in rheumatic children and controls

**Fig. 2:**
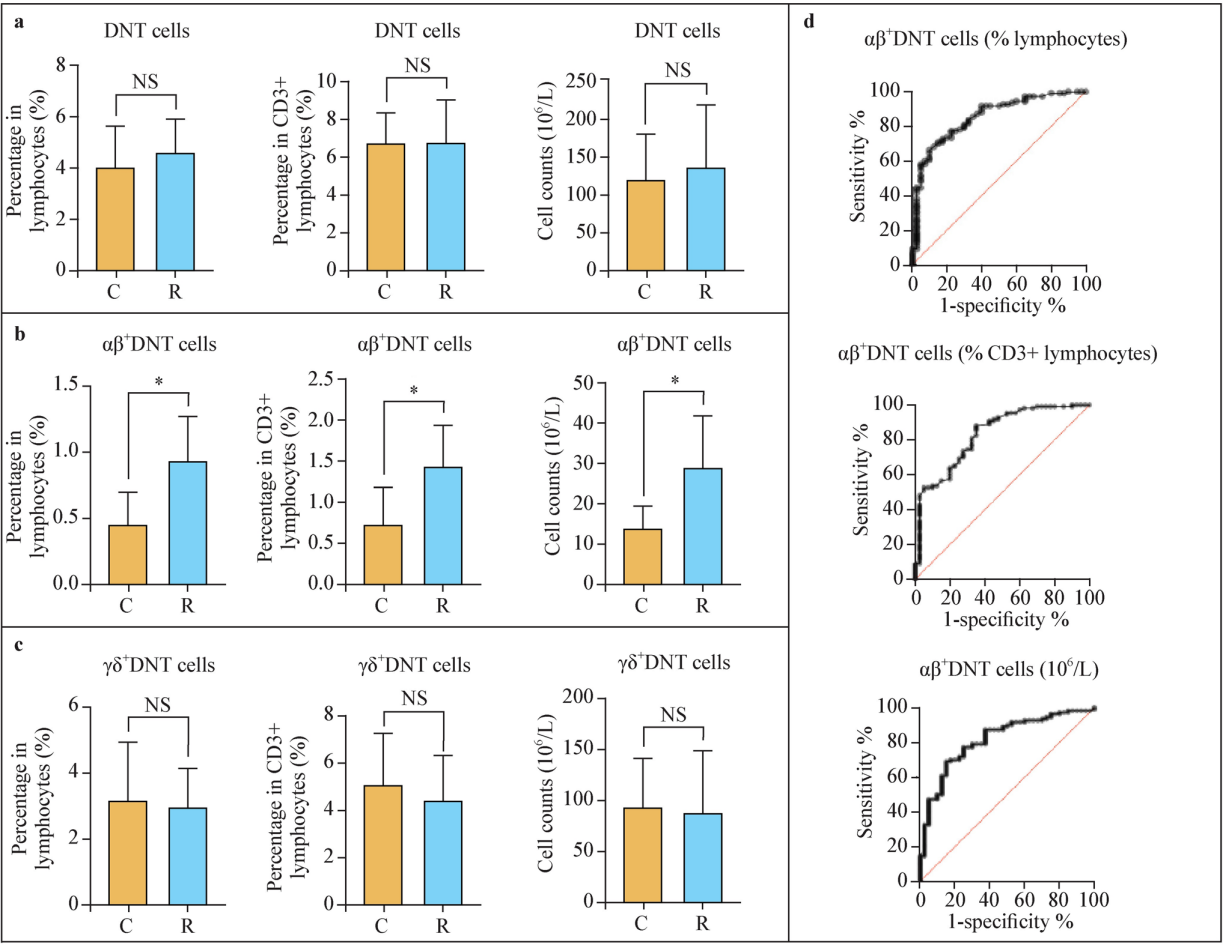
Comparison of DNT cells (**a**) and their two main subsets, αβ^+^DNT cells (**b**) and γδ + DNT cells (**c**) between rheumatic children and controls, along with ROC analysis related to the count of αβ.^+^DNT cells (**d**). *DNT* double-negative T, *TCR* T cell receptor, *ROC* receiver operating characteristic curve, *NS* not significant, * *P* < 0.001

Overall, the circulating DNT cell pool (defined as any T cell lacking both CD4 and CD8 markers) was not different in rheumatic children compared to the controls, as shown in Fig. 2a. However, when analyzed according to TCR type, it was evident that children with rheumatic disorders exhibited a significant increase in the number of αβ^+^DNT cells compared to controls (Fig. 2b), whereas this difference is not present in the γδ^+^DNT cell population (Fig. 2c).

In detail, regarding the αβ^+^DNT cell subset, this difference is observed regardless of the method used to count this population, whether as a percentage of total lymphocytes [*R*: 0.950 (0.730, 1.283)%; *C*: 0.445 (0.312, 0.707)%; *P* < 0.001], as a percentage of CD3^+^(T) lymphocytes [*R*: 1.440 (1.080, 1.935)%; *C*: 0.730 (0.5025, 1.195)%; *P* < 0.001], or as an absolute cell count [*R*: 28.92 (18.60, 42.14) × 10^6^/L; *C*: 13.70 (9.54, 19.20) × 10^6^/L; *P* < 0.001].

To further validate this significant difference in αβ^+^DNT cell numbers between rheumatic children and controls, corresponding ROC curves were generated for all three modes of cell counting: percentage of total lymphocytes [AUC = 0.8527, 95% confidence interval (CI) = 0.7851–0.9203; *P* < 0.001], percentage of CD3^+^(T) lymphocytes (AUC = 0.8314, 95% CI = 0.7574–0.9054; *P* < 0.0001), and absolute cell count (AUC = 0.8163, 95% CI = 0.7418–0.8907; *P* < 0.0001, Fig. 2d).

### Sub-analysis of αβ^+^ double-negative T cells in pediatric rheumatic diseases

**Fig. 3:**
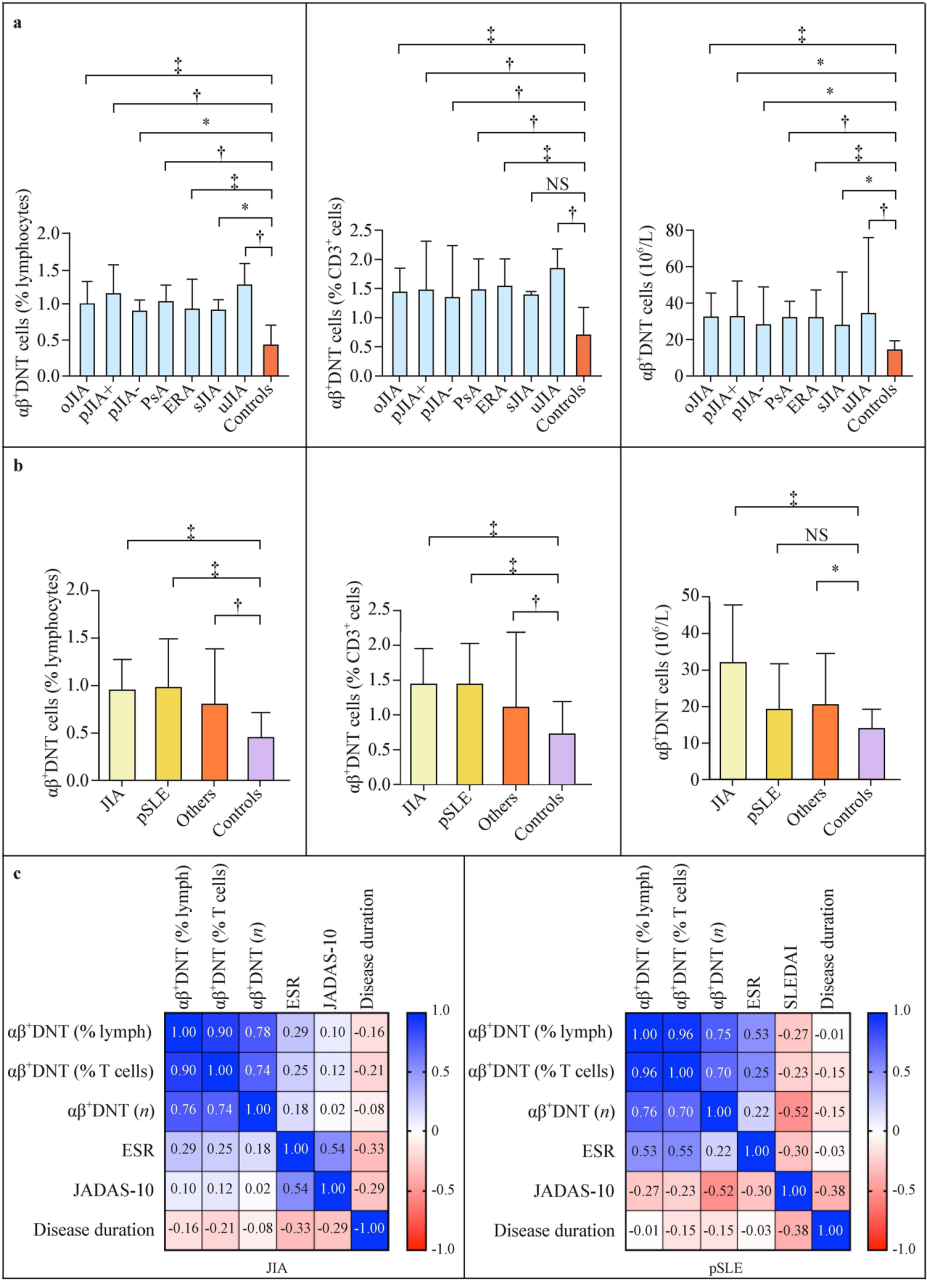
Comparison of αβ^+^DNT cells among different JIA subtypes (**a**) and rheumatic diseases (**b**), and covariation analysis of αβ.^+^DNT cells according to inflammatory markers and disease activity/stage parameters (**c**). *DNT* double-negative T, *JIA* juvenile idiopathic arthritis, *oJIA* oligoarticular JIA, pJIA polyarticular JIA, *PsA* psoriatic JIA, *ERA* enthesitis-related arthritis, *sJIA* systemic JIA, *uJIA* undifferentiated JIA, *pSLE* pediatric systemic lupus erythematosus, ESR *JADAS-10* juvenile arthritis disease activity index, SLEDAI SLE disease activity index, *NS* not significant, *P < 0.05, *†* *P* < 0.01, *‡* *P* < 0.001

JIA patients represent the majority of our study population in this preliminary research. Figure 3 shows that all JIA subtypes are characterized by a statistically significant increase in αβ^+^DNT cells compared to the controls, but no difference is present among the subtypes themselves. Moreover, no significant difference in circulating αβ^+^DNT cells was observed between JIA patients, pSLE patients, and children affected with other rheumatic diseases. However, αβ^+^DNT cells were increased in all these groups of rheumatic children compared to controls (Fig. 3b).

As mentioned, rheumatic children could be recruited at different stages of their disease course and were also subjected to variable therapeutic regimens. We analyzed the impact of these ongoing therapeutic regimens on the main lymphocyte subsets and, in detail, on the αβ^+^DNT cells (Table 3). We did not observe any significant differences based on the administration of steroids, cDMARDs, bDMARDs, or their combinations.

**Table 3:** Sub-analysis of αβ^+^DNT cell numbers according to the main drug regimens

Therapeutic regimens	αβ^+^DNT cells in lymphocytes (%)	αβ^+^DNT cells in CD3^+^ cells (%)	αβ^+^DNT cells ${(10}^{6}$/L)
Steroid drugs (SD)			
Yes	0.995 (0.75, 1.263)	1.460 (1.053, 1.965)	32.20 (18.72, 45.91)
No	0.875 (0.7275, 1.333)	1.360 (1.103, 1.855)	25.90 (17.64, 42.14)
* P *value	0.418	0.608	0.307
Biologics (bDMARDs)			
Yes	0.92 (0.625, 1.243)	1.370 (1.020, 2.025)	26.94 (18.51, 47.94)
No	0.98 (0.7625, 1.288)	1.445 (1.130, 1.828)	30.77 (18.71, 41.55)
* P *value	0.517	0.984	0.757
bDMARDs ± SD			
None (only cDMARDs) (*n* = 33)	0.88 (0.735, 1.195)	1.37 (1.095, 1.735)	26.57 (17.41, 38.61)
bDMARDs (*n* = 17)	0.86 (0.67, 1.53)	1.450 (1.085, 2.135)	25.07 (17.48, 49.28)
SD (*n* = 35)	1.06 (0.88, 1.29)	1.470 (1.190, 1.840)	33.32 (18.97, 40.97)
bDMARDs + SD (*n* = 25)	0.920 (0.610, 1.190)	1.33 (0.985, 2.010)	30.19 (18.58, 44.04)
* P* value	0.520	0.797	0.791
Main biologics			
Tocilizumab (*n* = 8)	0.89 (0.8125, 1.138)	1.390 (1.028, 1.553)	25.24 (21.68, 43.8)
Etanercept (*n* = 13)	1.180 (0.635, 1.685)	2.020 (1.125, 2.855)	31.47 (16.96, 59.02)
Adalimumab (*n* = 16)	0.815 (0.505, 1.318)	1.205 (0.7475, 2.045)	26.37 (18.00, 46.20)
* P* value	0.293	0.18	0.849

*DNT* double-negative T cell

Furthermore, no correlations were observed between αβ^+^DNT cell count and inflammatory parameters (such as ESR), or the disease activity index (JADAS-10) in JIA patients. In pSLE patients, we observed a trend of a moderate positive covariation of αβ^+^DNT cells with ESR when expressed as percentages of total lymphocytes or T cells (*r* = 0.525, *P* = 0.081; and *r* = 0.554, *P* = 0.065, respectively). Conversely, when αβ^+^DNT cells were expressed as an absolute cell count, they showed a trend of a negative correlation with pediatric SLE Disease Activity Index (pSLEDAI), though this did not reach statistical significance either (*r* =  − 0.523, *P* = 0.479), likely due to the small sample size. We also considered disease duration as an additional parameter related to disease stage, but did not find a correlation with the number of αβ^+^DNT cells. This covariation analysis is graphically summarized in Fig. 3c.

### Analysis of αβ^+^ double-negative T cells according to patients’ age

As mentioned earlier, rheumatic children and controls differ in age in this preliminary study. Therefore, to assess whether this age disparity may have impacted the significant difference in αβ^+^DNT cells number overall, we performed a sub-analysis within two main age groups: study participants aged 2–9 years (group 1) and those aged 10–17 years (group 2). This approach enabled us to compare rheumatic children and controls without any significant age differences within each group. Significant difference in the number of circulating αβ^+^DNT was found between children with rheumatic diseases and normal controls, which remained consistent in both age groups, regardless of the method used to count these cells (Table 4).

**Table 4:** Analysis of αβ^+^DNT cells and main parental populations according to the patients’ age

Characteristics	Control group	Rheumatic group	
**Group 1** (2–9 years)	**C** (*n* = 26)	**R** (*n* = 33)	**P-value**
**Gender **(M:F)	12:14	16:17	>0.9999
**Age **(Me, IQR)	5.25 (2.9, 7.525)	5.1 (3.4, 7.05)	0.5721
**DNTαβ**^**+**^ (% lymph)	**0.51 (0.32, 0.7575)**	**1.010 (0.845, 1.480)**	**<0.0001**
**DNTαβ**^**+**^ (% CD3^+^cells)	**0.88 (0.525, 1.218)**	**1.640 (1.200, 2.280)**	**<0.0001**
**DNTαβ**^**+**^ (10^6^/L)	**16.48 (12.61, 20.13)**	**40.08 (25.90, 73.49)**	**<0.0001**
**Lymphocytes **(10^6^/L)	3130 (2510, 4348)	4080 (2775, 5295)	0.0720
**CD3**^**+**^**cells** (10^6^/L)	2003 (1534, 2610)	2496 (1889, 3315)	0.0351
**αβ**^**+**^**CD3**^**+**^**cells ** (% lymph)	88.99 (86.62, 91.88)	90.34 (87.37, 93.15)	0.2040
**αβ**^**+**^**CD3**^**+**^**cells** (10^6^/L)	2852 (2241, 3698)	3515 (2884, 4860)	0.0549

**Group 2** (10–17 years)	**C** (*n* = 14)	**R** (*n* = 77)	**P-value**
**Gender **(M:F)	6:8	29:48	0.7698
**Age **(Me, IQR)	14.1 (11.45, 15.28)	14.3 (12.6, 16.11)	0.2904
**DNTαβ**^**+**^ (% lymph)	**0.37 (0.3075, 0.515)**	**0.91 (0.71, 1.200)**	**<0.0001**
**DNTαβ**^**+**^ (% CD3^+^cells)	**0.5950 (0.45, 0.9075)**	**1.390 (1.050, 1.815)**	**<0.0001**
**DNTαβ**^**+**^ (10^6^/L)	**9.670 (7.438, 12.13)**	**26.32 (17.26, 35.38)**	**<0.0001**
**Lymphocytes **(10^6^/L)	2470 (2273, 3095)	2770 (2300, 3395)	0.2365
**CD3**^**+**^**cells** (10^6^/L)	1519 (1366, 1990)	1982 (1512, 2298)	0.0572
**αβ**^**+**^**CD3**^**+**^**cells **(% lymph)	89.61 (86.41, 93.53)	92.36 (89.57, 94.41)	0.1769
**αβ**^**+**^**CD3**^**+**^**cells **(10^6^/L)	2242 (1985, 2909)	2548 (2111, 3031)	0.2053

The bold character further highlight at first glance that these parameters show a statistical significance between controls and rheumatic patients. It immediately shows that these parameteres keep their statistically significant differents in both age groups

*DNT* double-negative T cell, *M* male, *F* female, *Me* median, *IQR* interquartile range

This analysis further supports a real difference in the circulating αβ^+^DNT cell pool, despite the age difference between rheumatic patients and controls in the whole study population. Moreover, the comparison between groups 1 (younger children) and 2 (older children) in either rheumatic patients or controls did not show any differences in the circulating pool of αβ^+^DNT cells according to age (Supplementary Table 3), which further indicates no significant age-dependent variations of these cells. Finally, the linear regression analysis also supports this statement. Although a slight inverse relationship between age and αβ^+^DNT cell number was present in rheumatic patients (Supplementary Fig. 1), it was not pronounced enough to imply any analytical bias related to the age of the study participants. Moreover, these subtle age-related trends of αβ^+^DNT cells are likely attributable to the higher general lymphocyte counts in younger children within our study population (Table 4 and Supplementary Table 3), as it is also established in the pediatric age-related reference ranges for lymphocyte number, regardless of ethnicity [10–12].

Therefore, taken together, all these observations and analyses confirm the existence of a statistically significant increase in αβ^+^DNT cells in the bloodstream of children with rheumatic diseases, which is not attributable to the age difference between these groups.

## Discussion

The main finding emerging from the present study is that αβ^+^DNT cells in the peripheral blood are significantly increased in children with rheumatic disorders. However, this αβ^+^DNT cell expansion is not specific to any individual rheumatic disease since no differences were observed among JIA (including all JIA subtypes), pSLE, or other rheumatic patients, with all groups differing from controls. Moreover, our results also suggest that this increase in circulating αβ^+^DNT cells is not influenced by any specific therapeutic regimen (including steroids, cDMARDs, or bDMARDs). Finally, no significant association or correlation between the expansion of αβ^+^DNT cells and inflammatory parameters/disease activity was observed.

A recent systematic literature review suggested that DNT cells are variably and inconstantly increased in rheumatic children [9]. However, the immunophenotypic definition of DNT cells in these pediatric studies varied. Three studies identified the DNT cell population as CD4^−^CD8^−^ only [13–15], while two studies specified TCRαβ positivity on these CD4^−^CD8^−^ T cells [16, 17]. Additionally, three studies also assessed the expression of TCRγδ [18–20], as we did in the present research, but only one provided specific information on both αβ^+^DNT and γδ^+^DNT cells. The study by Kopitar et al. included children with multisystem inflammatory syndrome (MIS-C) and reported an increase in both DNT cell subsets compared to controls, with some differences according to the disease phase (acute and post-acute) [20]. Tarbox et al. and Mendonça et al. used the TCRγδ negativity on DNT cells to better define the αβ^+^DNT cell subset but did not provide specific information on the γδ^+^DNT cells [18, 19].

As mentioned, the present study clearly highlights the specific increase in the number of αβ^+^DNT cells in rheumatic children, without any concomitant alterations in γδ^+^DNT cells. This suggests a different regulation and/or role of these two subsets in pediatric rheumatic disorders, as supported by some experimental and clinical evidence [21, 22]. Interestingly, Pinheiro et al. investigated both αβ^+^DNT and γδ^+^DNT cells in patients with tuberculosis and observed that, unlike γδ^+^DNT cells, the number of circulating αβ^+^DNT cells was increased in infected people compared to healthy controls and positively correlated with disease severity [23]. Similarly, our findings showed this quantitative dichotomy between circulating γδ^+^DNT cells and αβ^+^DNT cells in rheumatic patients, but we could not observe any significant correlation with the inflammatory markers and/or disease activity. In this specific regard, the previous and aforementioned studies showed variable results in terms of correlation between DNT cells and inflammatory status and/or disease activity in pediatric patients [8, 9]. Indeed, only a couple of studies, both focused on children with pSLE, clearly analyzed this aspect but found no correlation between αβ^+^DNT cells and disease activity or other surrogated markers (such as ESR, C3 levels, and anti-dsDNA titer) [16, 17].

Overall, we observed an increase in circulating αβ^+^DNT cells across various types of rheumatic diseases, including JIA, pSLE, and others. Notably, no differences in αβ^+^DNT cell numbers were observed among the different JIA subtypes. This important observation suggests that the expansion of the circulating pool of αβ^+^DNT cells could be a nonspecific change related to the broader development of autoimmunity, thus not linked to any specific rheumatic disease. This hypothesis suggests that αβ^+^DNT cells might not have a precise immuno-pathological role in the rheumatic diseases studied. Instead, their numerical increase in the bloodstream could represent a “reactive” immunological phenomenon in the general context of autoimmunity, chronic inflammation, or sustained activation of the immune system. The fact that αβ^+^DNT cell numbers appear to be unaffected by the ongoing therapies in our patients further supports this hypothesis and is consistent with the findings by Pinheiro et al. in tuberculosis. Moreover, several studies have reported the expansion of the αβ^+^DNT cells or, in general, the circulating DNT cell pool in different infectious diseases, such as HIV infection [24–26], leishmaniasis [27], Chagas disease, [28, 29], and COVID-19 [30, 31].

This preliminary study has several limitations, including the small sample size, especially for non-JIA rheumatic diseases and the different ages between rheumatic and control children, though we attempted to lessen the impact of this factor on our results through an age-matched sub-analysis. Additionally, most patients were not recruited at the time of their diagnosis (i.e., before the start of any immunomodulatory therapy) and were subjected to different therapeutic regimens. Therefore, our findings should be validated by additional, larger studies, ideally incorporating a longitudinal analysis starting from disease onset.

In conclusion, αβ^+^DNT cells are significantly expanded in the blood of children with rheumatic diseases, unlike general DNT and γδ^+^DNT cells. However, this expansion does not appear to correlate with disease activity or therapeutic regimens. Our data suggests that the increase in circulating αβ^+^DNT cells could be a nonspecific marker of autoimmunity, unrelated to disease activity. However, larger studies are necessary to further investigate the significance of αβ^+^DNT cells in pediatric rheumatic diseases.

## Supplementary Information

Below is the link to the electronic supplementary material.

Supplementary file1 (PDF 606 KB)

## Data Availability

The datasets generated during and/or analyzed during the current study are available from the corresponding author on reasonable request.
