# Supplementary material for: Nonspecific increase of αβTCR+ double-negative T cells in pediatric rheumatic diseases
Source: World J Pediatr. 2024 Nov 28;20(12):1283–92. doi: 10.1007/s12519-024-00854-7 (PMC11634929; doi:10.1007/s12519-024-00854-7)
Supplement: Supplementary file 1 — Supplementary file1 (PDF 606 KB) [file 12519_2024_854_MOESM1_ESM.pdf]

SUPPLEMENTARY MATERIAL

Supplementary Table 1. General hematological and inflammatory parameters.

Supplementary Table 2. General lymphocyte subpopulations.

Supplementary Table 3. Statistical comparison of αβ+DNT cells and main parental populations between younger and older children in both controls (C) and rheumatic patients (R), respectively; and covariation analysis between αβ+DNT cells and main parental populations according to the patients' age.

Supplementary Figure 1. Linear regression analysis between αβ+DNT cell counts and patients' age, considering all study population (R+C), only controls (C), and only rheumatic children (R).

Supplementary Table 1. General hematological and inflammatory parameters.

**Characteristic**	**Patients (n=110)**	**Controls (n=40)**	**p-value[1]**
White Blood cells (10^9/L)	7.350 (5.958, 8.883)	7.260 (6.375, 8.493)	0.9637
Hemoglobin (g/L)	131.5 (121.8, 138.0)	130.0 (124.3, 136.0)	0.7133
Neutrophils (%)	44.7 (33.38, 52.63)	45.6 (36.95, 58.38)	0.5027
Neutrophils (10^9/L)	3.090 (2.203, 4.228)	3.155 (2.088, 4.550)	0.7613
Lymphocytes (%)	42.75 (34.9, 54.18)	44.15 (31.30, 49.38)	0.6136
Lymphocytes (10^9/L)	***2.955 (2.510, 4.070)***	***3.025 (2.293, 3.435)***	***0.4515***
Monocytes (%)	8.00 (6.375, 9.725)	7.450 (5.950, 8.90)	0.1916
Mononocytes (10^9/L)	0.605 (0.480, 0.720)	0.540 (0.415, 0.690)	0.1444
Eosinophils (%)	2.400 (1.475, 3.725)	2.750 (1.525, 4.500)	0.4238
Eosinophils (10^9/L)	0.170 (0.10, 0.290)	0.170 (0.120, 0.360)	0.6016
Basophils (%)	0.600 (0.400, 0.700)	0.500 (0.325, 0.775)	0.4635
Basophils (10^9/L)	0.040 (0.030, 0.060)	0.040 (0.030, 0.0575)	0.7373
ESR (mm/h)	***15.00 (6.500, 24.50)***	***7.00 (4.250, 11.50)***	***0.0004***

Supplementary Table 2. General lymphocyte subpopulations.

Characteristics	Rheumatic (n=110)	Controls (n=40)	p-value
**T cells** (%)	73.09 (66.58, 77.74)	65.99 (58.88, 70.88)	<0.0001
**T cells** (10^6/L)	2135 (1706, 2670)	1836 (1439, 2312)	0.0196
**$CD4^+$ T cells** (%)	53.43 (45.08, 59.54)	59.3 (53.07, 63.98)	0.0005
**$CD4^+$ T cells** (10^6/L)	1601 (1248, 2030)	1599 (1332, 2211)	0.5322
**$CD8^+$ T cells** (%)	38.24 (33.54, 45.654)	38.49 (28.09, 38.49)	0.0002
**$CD8^+$ T cells** (10^6/L)	1135 (900.4, 1638)	896.3 (710.1, 1312)	0.0080
**CD4/CD8**	1.420 (1.010, 1.780)	1.783 (1.410, 2.209)	0.0001
**NK cells** (%)	9.475 (6.825, 13.73)	14.73 (10.58, 20.47)	<0.0001
**NK cells** (10^6/L)	304.3 (178.7, 436.8)	472.5 (269.3, 727.9)	0.0005
**B cells** (%)	14.36 (9.673, 19.45)	16.15 (13.55, 23.43)	0.0265
**B cells** (10^6/L)	431.9 (237.5, 676.5)	473.1 (332.0, 797.1)	0.2562

Supplementary Table 3. Statistical comparison of αβ^{+}DNT cells and main parental populations between younger and older children in both controls (C) and rheumatic patients (R), respectively; and covariation analysis between αβ+DNT cells and main parental populations according to the patients' age.

**Group 1 vs Group 2** (Mann-Whitney test)			
		**C**	**R**
**Gender** (p-value)		>0.9999	0.2997
**Age** (p-value)		<0.0001	<0.0001
**DNTαβ^{+}** $_{\%\ lymph}$ (p-value)		*0.1601*	*0.0719*
**DNTαβ^{+}** $_{\%\ CD3+\ cells}$ (p-value)		*0.3285*	*0.0409*
**DNTαβ^{+}** $_{10\text{^}6/L}$ (p-value)		*0.0013*	*<0.0001*
**Lymph** $_{10\text{^}6/L}$ (p-value)		0.0558	<0.0001
**CD3^{+} cells** $_{10\text{^}6/L}$ (p-value)		0.0416	<0.0001
**αβ^{+}CD3^{+} cells** $_{\%\ lymph}$ (p-value)		0.3918	0.0423
**αβ^{+}CD3^{+} cells** $_{10\text{^}6/L}$ (p-value)		0.0670	<0.0001
**(All) Controls vs (All) Rheumatic patients** (Covariation – Spearman coefficient)			
**DNTαβ^{+}** (% lymph)		**C**	**R**
**Age**	r	0.010	-0.157
	p	0.950	0.102
**DNTαβ^{+}** (% CD3^{+} cells)		**C**	**R**
**Age**	r	0.027	-0.189
	p	0.867	0.048
**DNTαβ^{+}** (10^6/L)		**C**	**R**
**Age**	r	-0.355	-0.317
	p	0.024	0.007
**Lymph** (10^6/L)		**C**	**R**
**Age**	r	-0.450	-0.320
	p	0.004	0.001
**CD3^{+} cells** (10^6/L)		**C**	**R**
**Age**	r	-0.430	-0.270
	p	0.005	0.004
**αβ^{+}CD3^{+} cells** (% lymph)		**C**	**R**
**Age**	r	0.1	0.21
	p	0.527	0.024
**αβ^{+}CD3^{+} cells** (10^6/L)		**C**	**R**
**Age**	r	-0.440	-0.300
	p	0.005	0.001

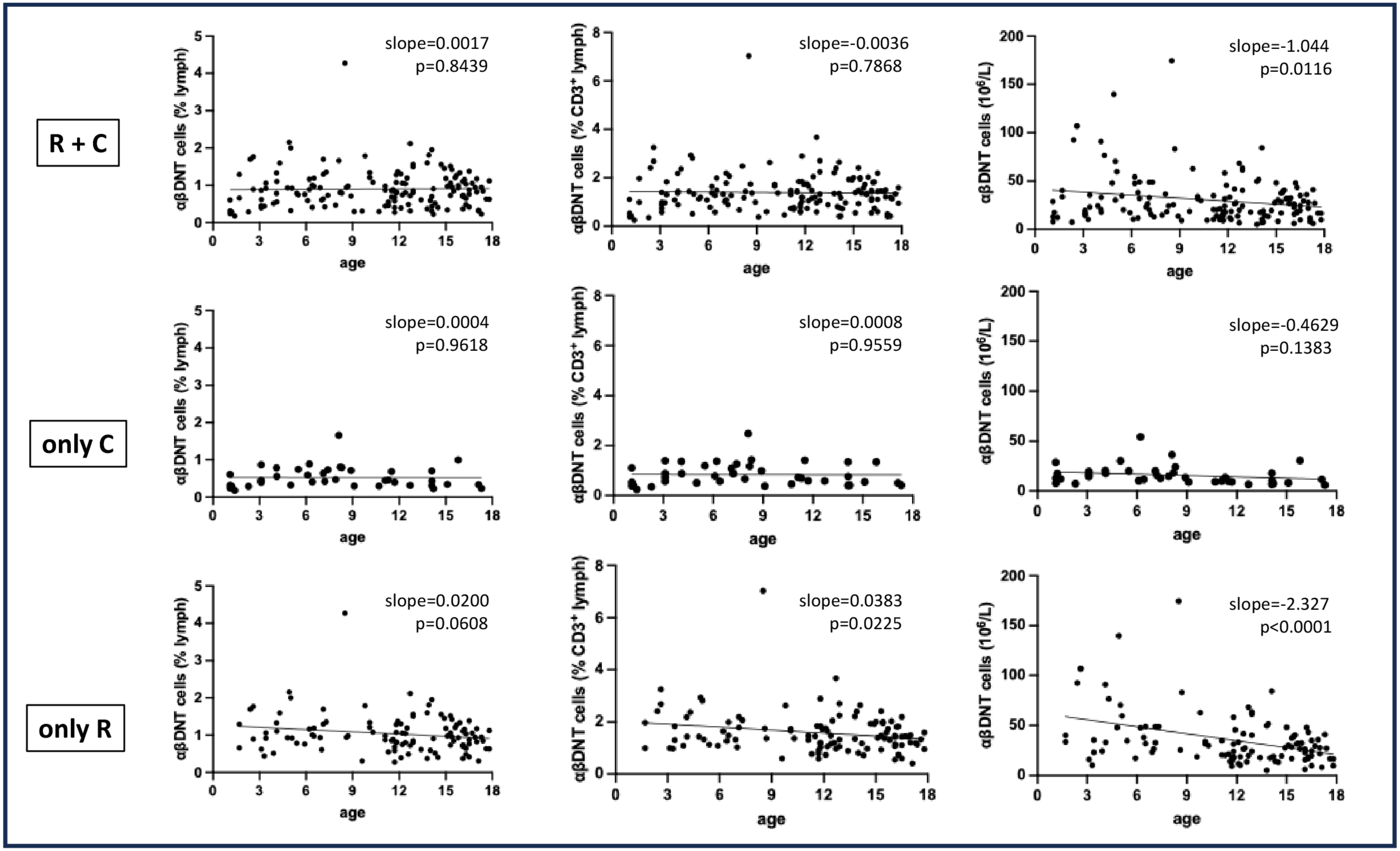
R + C
only C
only R
αβDNT cells (% lymph)
αβDNT cells (% CD3⁺ lymph)
αβDNT cells (10⁶/L)
age
slope=0.0017
p=0.8439
slope=-0.0036
p=0.7868
slope=-1.044
p=0.0116
slope=0.0004
p=0.9618
slope=0.0008
p=0.9559
slope=-0.4629
p=0.1383
slope=0.0200
p=0.0608
slope=0.0383
p=0.0225
slope=-2.327
p<0.0001
